# Nicotine abolishes memory‐related synaptic strengthening and promotes synaptic depression in the neurogenic dentate gyrus of miR‐132/212 knockout mice

**DOI:** 10.1111/adb.12905

**Published:** 2020-04-15

**Authors:** Tamara Stojanovic, Hannah Benes, Amena Awad, Daniel Bormann, Francisco J. Monje

**Affiliations:** ^1^ Center for Physiology and Pharmacology, Department of Neurophysiology and Neuropharmacology Medical University of Vienna Vienna Austria

**Keywords:** hippocampus, miR‐132/212, nicotine

## Abstract

Micro‐RNAs (miRNAs) are highly evolutionarily conserved short‐length/noncoding RNA molecules that modulate a wide range of cellular functions in many cell types by regulating the expression of a variety of targeted genes. miRNAs have also recently emerged as key regulators of neuronal genes mediating the effects of psychostimulant drugs and memory‐related neuroplasticity processes. Smoking is a predominant addictive behaviour associated with millions of deaths worldwide, and nicotine is a potent natural psychoactive agonist of cholinergic receptors, highly abundant in cigarettes. The influence of miRNAs modulation on cholinergic signalling in the nervous system remains however poorly explored. Using miRNA knockout mice and biochemical, electrophysiological and pharmacological approaches, we examined the effects of miR‐132/212 gene disruption on the levels of hippocampal nicotinic acetylcholine receptors, total ERK and phosphorylated ERK (pERK) and MeCP2 protein levels, and studied the impact of nicotine stimulation on hippocampal synaptic transmission and synaptic depression and strengthening. miR‐132/212 deletion significantly altered α7‐nAChR and pERK protein levels, but not total ERK or MeCP2, and resulted in both exacerbated synaptic depression and virtually abolished memory‐related synaptic strengthening upon nicotine stimulation. These observations reveal a functional miRNAs/nicotinergic signalling interplay critical for nicotinic‐receptor expression and neuroplasticity in brain structures relevant for drug addiction and learning and memory functions.

## Graphical abstract

Micro‐RNAs are key regulators of neuronal genes mediating the effects of psychostimulant drugs and neuroplasticity. Using miRNA knockout mice and biochemical, electrophysiological and pharmacological approaches, this work describes that miR‐132/212 gene deletion alters molecular elements from the cholinergic signalling pathway (α7‐nAChR and pERK protein levels) and not only exacerbates synaptic depression but also virtually abolishes memory‐related synaptic strengthening upon nicotine stimulation.

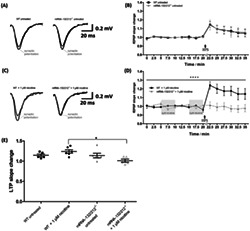

## 1 INTRODUCTION

Micro‐RNAs (miRNAs) are short‐length noncoding RNAs that can act as powerful post‐transcriptional regulators of gene expression^1^ by inducing either degradation or blockade of translation of selectively targeted gene products.^2^, ^3^, ^4^, ^5^, ^6^, ^7^ miRNAs are highly evolutionarily conserved in the plant^8^ and animal kingdoms.^9^, ^10^ In mammals, miRNAs have been involved in the regulation of more than 50% of all genes known to be encoding for the formation of proteins.^11^ miRNAs indeed modulate a wide‐ranging variety of cellular processes, spanning from early‐to‐late stages of embryonic development to basic metabolic functions, homeostasis, cellular proliferation and differentiation and secretion.^12^, ^13^, ^14^, ^15^, ^16^ miRNAs play also critical roles in the regulation of the neuronal function, a characteristic highly conserved from an evolutionary perspective as demonstrated in organisms such as zebra fish, mice and humans.^14^, ^17^, ^18^, ^19^, ^20^ For example, miRNAs are critical for neural development^21^ and adult neurogenesis,^14^, ^18^, ^19^ and memory‐related synaptic plasticity.^22^, ^23^ Dysregulation of miRNAs activity is further associated with a wide variety of severely debilitating human neurodegenerative conditions such as epilepsy,^24^, ^25^, ^26^ Alzheimer's^27^, ^28^, ^29^, ^30^, ^31^, ^32^, ^33^, ^34^, ^35^ and Parkinson's diseases.^11^

The miR‐132/212 family of miRNAs has been proposed as a key regulator of spinal cord development,^36^ embryonic stem cell differentiation into neurons^37^ and memory‐related neuroplasticity.^22^, ^38^ Dysregulation of miR‐132 has been further associated with the genesis and worsening of epilepsy, Alzheimer's and Parkinson's diseases.^39^ Little remains known, however, about the involvement of miR‐132/212 in the regulation of synaptic functions in neuronal circuits critical for both learning and memory and drug addiction.

Acetylcholine (Ach)^40^, ^41^ is a neurotransmitter highly active both in the peripheral and in the central nervous systems, where it is known to act as a key regulator of neuroplasticity and learning and memory processes. Ach interacts with—and regulates—both muscarinic and nicotinic receptors and regulates hippocampal synaptic plasticity and learning and memory processes.^42^, ^43^, ^44^, ^45^, ^46^, ^47^, ^48^, ^49^, ^50^, ^51^, ^52^, ^53^, ^54^, ^55^ Whereas both muscarinergic‐^56^ and nicotinergic‐mediated signalling are thus of pivotal importance for the regulation of the brain cognitive function, the predominance of tobacco over mushrooms as the worldwide preferred substance of abuse is disproportionally superior.^57^, ^58^ We therefore here focused on nicotinergic‐related functions in the mammalian hippocampus. Nicotinic acetylcholine receptors (nAChrs)^59^, ^60^ comprise a group of ligand‐gated ion channels abundant in neurons of the central nervous system^61^ that modulate neuroplasticity and memory functions and are importantly implicated in tobacco‐derived nicotine addiction.^62^, ^63^, ^64^, ^65^ However, although both miRNAs‐mediated and nicotinergic signalling have been independently described as critical regulators of neuroplasticity, learning and memory and addiction‐related functions, the functional crosstalk between these two pivotal mechanisms of neuronal regulation in brain neuronal circuits have remained virtually unexplored.

Using the mouse as experimental model, this work specifically examined how the knockout (KO) of the gene encoding for miR‐132/212 influences the neuroplasticity responses to nicotine stimulation at the hippocampal neurogenic dentate gyrus. Our results provide, to the best of our knowledge, the first biochemical and functional experimental evidence in support of a role of miR‐132/212 in the effects of nicotine on memory‐related synaptic plasticity in the mammalian hippocampus, a brain region critical for memory storage and importantly implicated in the neurobiology of tobacco‐derived nicotine addiction. These results broaden our understanding of the molecular mechanisms influencing the neuronal responses to addictive substances and unveil the miR‐132/212 family of miRNAs as putative candidate targets to mediate in the capability of nicotine to alter the function of neuronal circuits underlying memory formation of drug addictions.

## 2 MATERIALS AND METHODS

### 2.1 Animals

Experiments were conducted using male wild‐type (WT) C57Bl/6 and genetically modified miRNA‐132/212 KO (miRNA‐132/212^−/−^) mice in a C57Bl/6 background (15–20 weeks old). Generation of the miRNA‐132/212^−/−^ used here is described in Remenyi et al.^38^ Littermates with confirmed WT genotype were used as controls. All mice were housed in groups of three to five mice per standard Plexiglas housing cage in a colony room with controlled temperature (22 ± 2)°C and light (200 ± 20) lx conditions. Food and water were provided ad libitum. The illumination was controlled automatically on a 12‐h light/dark cycle with light period starting at 6:00 am. The experimental study of WT and miRNA‐132/212^−/−^ brain neuronal functions followed Bundesministerium für Wissenschaft und Forschung of Austria directives (BMWF‐66.009/0200‐WF/V/3b/2016). FM holds a GV‐SOLAS/FELASA‐equivalent training accreditation in the use of laboratory animals in experimental sciences issued by the University of Veterinary Medicine of Vienna Austria. TS successfully completed the Course on Laboratory Animal Science, organized by the Center for Biomedical Research, Medical University of Vienna, Austria. Maintenance and managements of experimental animals were conducted under advice from two expert veterinarians and accordance with the ARRIVE guidelines and the U.K. Animals (Scientific Procedures Act, 1986, and associated guidelines, EU Directive 2010/63/EU for animal experiments).

### 2.2 Immunoblotting

Brain extraction and whole hippocampi isolation from miRNA‐132/212^−/−^ and WT male mice (*n* = 6 animals per group) were conducted according to methods established in our laboratory as described before,^66^, ^67^, ^68^ with minor modifications.^69^ Animals were euthanized by quick cervical dislocation, followed by a swift sharp‐blade decapitation. Brains were rapidly extracted and submerged in an ice‐cold artificial cerebrospinal fluid (aCSF) with pH adjusted to 7.4. Hippocampi were carefully isolated, and cerebellum was dissected. Hippocampi and the rest of the brain were individually sampled, frozen in liquid nitrogen and immediately stored at −80°C until further analysis. Brain tissues were further homogenized in lysis buffer (50‐mM Tris, 150‐mM NaCl, 1% TritonX‐100 and 5‐mM EDTA) and 1:100 Protease and Phosphatase Inhibitor Cocktail (PIC, Thermo Scientific, Germany) and incubated overnight at 4°C on a tube rotator. Following overnight incubation, lysates were centrifuged for 5 min at 12 000 *g* (Heraeus Fresco 17 R‐134a, Thermo Scientific, Germany) at 4°C. Supernatants (25 μg of proteins per lane) were electrophoresed on a 10% sodium dodecyl sulfate (SDS)‐polyacrylamide gel and transferred to poly(vinylidene difluoride) (PVDF) membranes (Immobilon P Transfer Membrane, Merck Millipore). The membranes were blocked in 5% bovine serum albumin (BSA) (A8022, Sigma, Germany) and incubated overnight in primary antibodies α7‐nAChR (1:1000; Cat Nr. ab10096 Abcam), ERK 1:1000 (Cat Nr. 14‐9108‐82, Thermo Fisher Scientific eBioscience™), pERK (1:1000; Cat No. 4376 Cell Signalling) and MeCP2 (1:1000; Cat. No. 3456 Cell Signalling) diluted in 1% BSA solution in Tris‐buffered saline + Tween 20 (Polysorbate 20) (TBST). Goat anti‐rabbit was used as secondary antibody (1:3000; Cat No. 7074S Cell Signalling). GAPDH (1:3000; Cat No. MA5‐15738 Thermo Fisher Scientific) was used as a loading control. Blots were developed using the electrochemical luminescence (ECL) reagent (1705061, Bio‐Rad). Proteins were visualized by FluorChem HD2 system (Alpha Innotech, San Leandro, CA, USA), and levels quantified by densitometry using the ImageJ software (http://rsbweb.nih.gov/ij/).

### 2.3 Electrophysiology

#### 2.3.1 Hippocampal slices preparation

Acute hippocampal slices were prepared from miRNA‐132/212^−/−^ and littermate WT male mice (*n* = 5–6 animals per group) according to a previously published protocols,^66^, ^67^, ^68^, ^70^ with minor modifications. Following quick cervical dislocation and swift sharp‐blade decapitation, brains were extracted by a midline incision in the occipital bone starting at the foramen magnum extending to the sutura frontalis. The parietal bones were folded to the side, and brain was gently removed, using a spatula, and submerged in an ice‐cold aCSF solution containing (in mM) 125 NaCl, 2.5 KCl, 20 NaHCO_3_, 2.5 CaCl_2_, 1 MgCl_2_, 25 d‐glucose and 1 NaH_2_PO_4_ (pH 7.4). Coronal hippocampal slices were cut using a vibrating microtome (7000smz‐2, Campden Instruments Ltd., Loughborough, UK) at a frequency of 90 Hz, an amplitude of 0.75 mm and speed of 0.12 mm/s. During cutting, slices remained continually submerged in ice‐cold aCSF solution bubbled with a carbogen gas mixture (95% O_2_/5% CO_2_). After cutting, hemispheres were separated, and slices transferred to a customized recovery chamber containing carbogenated aCSF at 28°C where slices were left to rest for at least 1 h before electrophysiological recording.

#### 2.3.2 Extracellular recordings

**FIGURE 1:**
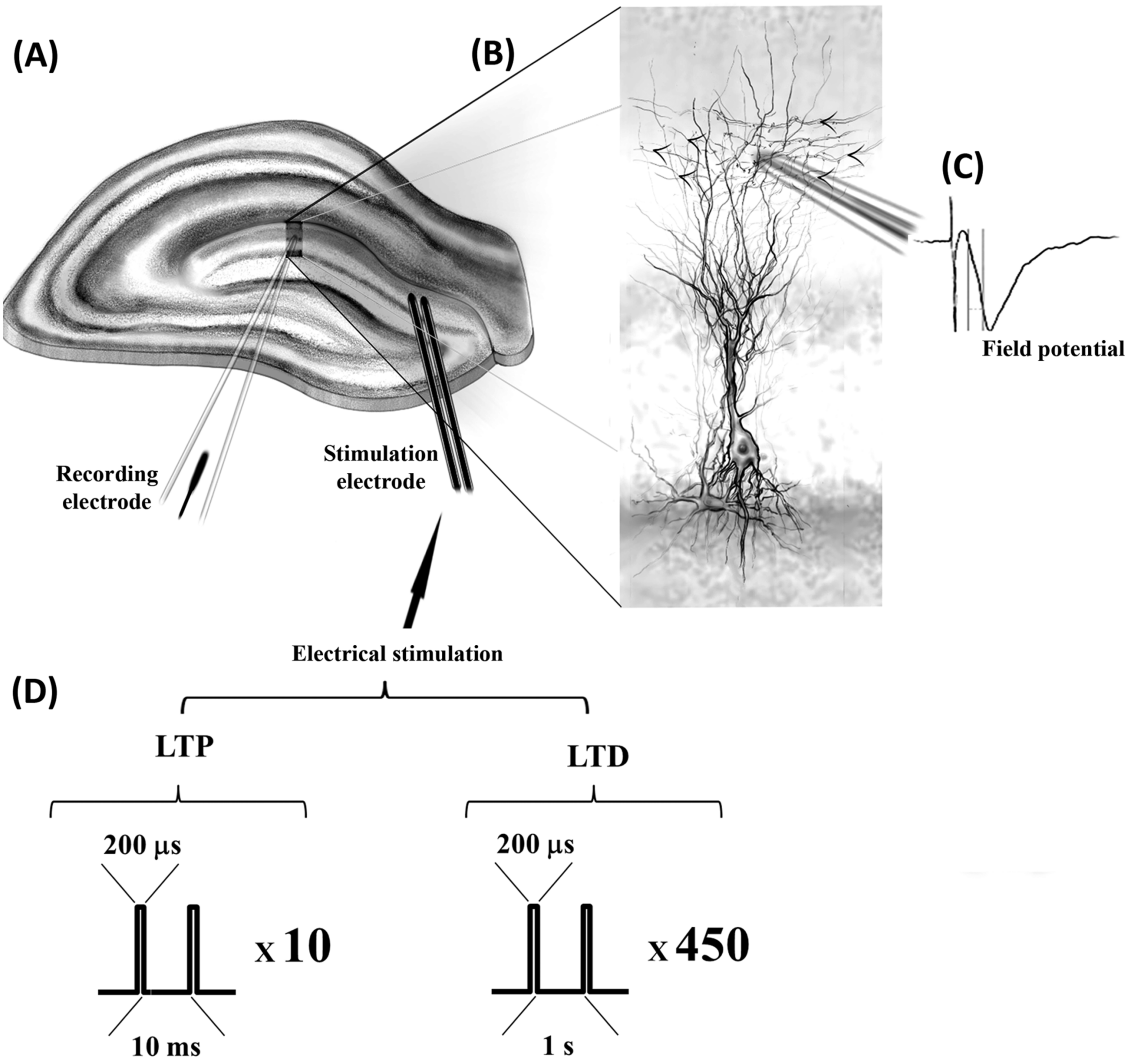
Schematic representation of the electrophysiological experimental setting. A, Diagram of a hippocampal slice with the positioning of the stimulating and recording electrodes at the neurogenic dentate gyrus. Simulating electrodes are positioned upstream of the middle molecular layer to stimulate the medial perforant path. B, The inset depicts a recording electrode positioned at the dendritic layer of neuronal granule cells, with arrows indicating the direction of the inputs from the perforant path. C, A representation of a recorded extracellular field potential is shown to the right. D, Diagrams of the protocols of electrical stimulation delivered through the stimulation electrodes are described for long‐term potentiation (LTP) and for long‐term depression (LTD) (see also Section 2)

Individual slices were transferred to a low volume submerged recording chamber perfused with a constant flow (2–3 mL/min) of carbogenated aCSF solution maintained at the room temperature. Evoked field excitatory postsynaptic potentials (fEPSPs) were recorded using borosilicate glass pipettes prepared in a horizontal puller (Sutter Instrument, Novato, CA) and yielding tip resistance of (3 ± 1) MΩ when filled with aCSF. Electrical stimulation was delivered via customized Teflon‐coated tungsten wire bipolar stimulating electrodes isolated to the tip (~50‐μm diameter tip). Recordings of fEPSPs were obtained from the dentate gyri middle molecular cell layer upon stimulation of the medial perforant pathway (MPP) as previously described by our group and others.^68^, ^71^ Standard input/output (I/O) curves were generated by plotting the data obtained for different series of fEPSP field slopes (by using linear fit on each trace starting from 20% and ending at 80% of the decaying phase) as measured in response to increasing square pulses (200 μs, 15‐s interpulse interval, 0–9 V and 1‐V increments) of electrical stimulation. Input values eliciting 40%–50% (for synaptic potentiation [LTP] measurements) or 70%–80% (for synaptic depression [LTD] measurements) of the maximum field‐slope response were used to examine basal synaptic transmission and to deliver high‐ or low‐frequency stimulation to induce potentiation or depression, respectively (Figure 1). Different electrical stimulation protocols have been successfully implemented in vivo as well as in hippocampal slices in order to induce LTP and LTD.^67^, ^72^, ^73^, ^74^ While several laboratories concomitantly studying both LTP and LTD in slices have reported the use of the same number of pulses for evoking LTP and LTD but with different frequency protocols, other groups have followed a different approach examining LTP and LTD with different frequencies and different number of pulses.^75^, ^76^, ^77^, ^78^, ^79^, ^80^, ^81^, ^82^, ^83^, ^84^ Several groups, including ours, have reported consistent depression of synaptic responses in hippocampal synapses after the delivery of a low‐frequency stimulation protocol consisting of 900 pulses at a frequency of 1 Hz.^77^, ^85^, ^86^, ^87^ We therefore here induced LTD by delivering 900 paired pulses (200 μs/pulse, 40‐ms interpulse interval) at a frequency of 1 Hz (Figure 1). LTP, on the other hand, can be also induced via a variety of several different types of electrical stimulation protocols in mouse hippocampal slices, with magnitude and durations of the responses strongly depending on the number and frequency of delivered pulses.^74^, ^80^ A discrete pulse of high‐frequency electrical stimulation at 100 Hz for only 1 s, for example, is capable of inducing transitory synaptic potentiation for more than 30 min in both CA3‐Schaffer collateral to CA1 and dentate gyrus synapses, whereas increased stimulation (number of pulses and different frequencies of presentation) can result in stronger and more long‐lasting responses.^74^, ^81^, ^88^ These relationships between the characteristics of the electrical stimulation protocols and the magnitude and duration of the synaptic responses have been also observed in in vitro reconstituted synapses from *Aplysia*, where it has been also demonstrated that brief electrical stimulation protocols can generate significantly enhanced and enduring synaptic responses when presented in combination with neurotransmitters stimulation.^89^, ^90^, ^91^, ^92^

Previous reports examining the effects of nicotine on LTP have further reported that a weak tetanic stimulation (20 pulses at 100 Hz) is capable of inducing stable LTP in the hippocampal CA1 region).^93^ Studies of miR‐132/212 KO mice have also demonstrated significant differences in hippocampal LTP in these animals (as examined using hippocampal slices) depending upon the stimulation protocol, compared with WT controls.^38^ In miR‐132/212 KO mice, however, the impact of neurotransmitter stimulation on hippocampal synaptic plasticity upon presentation of short‐term forms of potentiation‐inducing electrical stimulation has not been described. Therefore, LTP was here induced by delivering only 20 pulses (200 μs/pulse) of electrical stimulation at a frequency of 100 Hz alone and in combination with nicotine.

Paired‐pulse inhibition (PPI) field responses were evoked by two consecutive pulses of electrical stimulation (eliciting 40%–50% of maximum amplitude) with increasing interpulse intervals of 20, 40, 60, 80 and 100 ms. Baseline, PPI and post‐high and low‐frequency stimulation responses were recorded at 0.033 Hz. Stimulation protocols were delivered at baseline stimulation intensities. LTP and LTD were determined by analysing changes in the decaying phase of fEPSP slopes after low/high‐frequency stimulation, normalized to baseline. Data were averaged within a 2.5‐min interval (five consecutive sweeps), further averaged within groups and compared between miRNA‐132/212^−/−^ and WT mice with or without nicotine presence in the recording chamber. For specific experiments (Section 3), slices were simulated twice for 5 min (with a 5‐min interval) with aCSF solution containing 1‐μM nicotine (N5260, Sigma‐Aldrich) as used in independent experimental settings described before.^53^, ^94^, ^95^ Electrical stimulation was generated from an ISO‐STIM 01D (NPI Electronics, Tamm, Germany). An AxoClamp‐2B amplifier, Digidata‐1440 interface (Axon Instruments, MolecularDevices, Berkshire, UK) and the pClamp‐11 software (Molecular Devices, Berkshire, UK) were used for the acquisition and analysis of data.

### 2.4 Statistical analysis

Statistical analyses were done using GraphPad Prism Software, Version 7.0 (San Diego, CA, USA). The normality test D'Agostino's K^2^ test was used prior to any statistical analyses, to determine the sample data distribution. Two‐sided unpaired Student's *t* test was used to determine differences of data sets obtained from two experimental groups. Statistical analysis involving more than two experimental groups was assessed using analysis of variances (ANOVA) as appropriate, specifically repeated‐measures ANOVA (or mixed model ANOVA) followed by Bonferroni's test to account for multiple comparisons. *P* values and the number of samples used (*n*) are presented in the figure legends. All data are expressed as means ± standard error. Significance level (*α*) was set at 0.05 in all analyses.

## 3 RESULTS

### 3.1 Unaltered basal synaptic transmission and PPI in miRNA‐132/212^−/−^ mouse dentate gyrus

**FIGURE 2:**
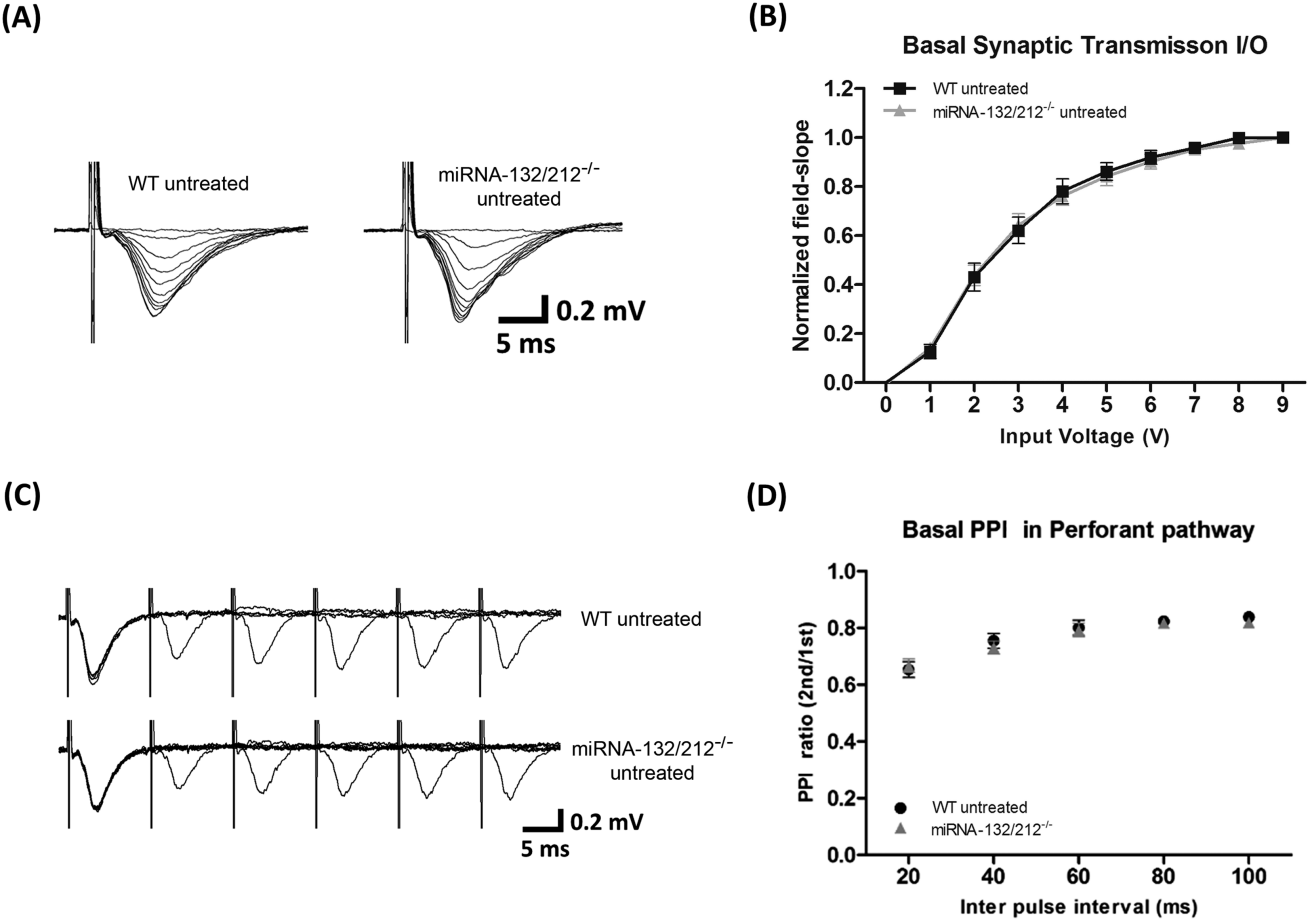
miRNA‐132/212 gene disruption does not disturb basal synaptic transmission or paired‐pulse inhibition (PPI) in the hippocampal dentate gyrus. A, Representative raw traces of extracellular field potentials obtained from hippocampal slices from untreated control wild‐type mice and from slices from untreated miRNA‐132/212^−/−^ in response to input/output (I/O) stimulation protocols (Section 2) with no apparent differences. B, Normalized field slope averaged values versus the increasing pulses of input stimulation voltages from untreated control wild‐type mice (*n* = 10) and from slices from untreated miRNA‐132/212^−/−^ (*n* = 11) as generated from the application of I/O stimulation protocols (Section 2). No statistical differences between genotypes were detected. C, Comparative examinations of field potentials recorded during PPI protocols in adult dentate gyrus of slices obtained from untreated wild‐type (*n* = 10) and untreated miRNA‐132/212^−/−^ (*n* = 11) mice. Data revealed no statistically significant differences between groups. D, Normalized and averaged PPI ratios (second pulse/first pulse) versus different interpulse intervals showing no differences between groups. Please see details for the statistics on the main text

To examine the effects of miRNA‐132/212 gene disruption on the properties of synaptic transmission and neuroplasticity, we performed ex vivo electrophysiological recordings in the dentate gyrus middle granule cell layer (Figure 1) of WT and miRNA‐132/212^−/−^ mice after the application of a series of standardized protocols of electrical stimulation (Section 2). Recordings were conducted in the absence and presence of 1‐μM nicotine, and neuroplasticity responses to high‐ and low‐frequency electrical stimulation (known to induce synaptic strengthening and depression, respectively) were examined by recordings of extracellular field potentials (Section 2). We first examined the properties of basal synaptic transmission by generating I/O curves (Section 2) in hippocampal slices from miRNA‐132/212^−/−^ mice and control WT littermates. Recordings obtained from miRNA‐132/212^−/−^ mice gave rise to I/O curves that were indistinguishable from those obtained from the WT littermate counterparts (Figure 2A,B) indicating intact basal synaptic transmission in the miRNA‐132/212^−/−^. Two‐way repeated measures ANOVA showed main significant effect of input voltage (^****^ *P* < 0.0001, *F*_1.17,10.50_ = 570.8) but no significant effect of genotype (^ns^ *P* = 0.8887, *F*_1,9_ = 0.02073). We next examined the properties of PPI, a de novo protein synthesis‐independent short‐term form of synaptic plasticity typically detectable in the dentate gyrus granule cells upon stimulation of the perforant path, a region involved in learning and memory and epilepsy‐related processes.^96^, ^97^, ^98^, ^99^ Recordings from untreated miRNA‐132/212^−/−^ mice slices presented with PPI responses statistically comparable with those measured in slices from WT littermate controls (Figure 2C,D) indicating no change in presynaptic dependent plasticity upon deletion of mi‐RNA132/212 (two‐way repeated measures ANOVA, significant effect of interpulse interval ^****^ *P* < 0.0001, *F*_1.77,15.91_ = 66.97, and no significant effect of genotype ^ns^ *P* = 0.7479, *F*_1,9_ = 0.1098).

### 3.2 Nicotine stimulation abolishes dentate gyrus synaptic potentiation in miRNA‐132/212^−/−^ mouse

**FIGURE 3:**
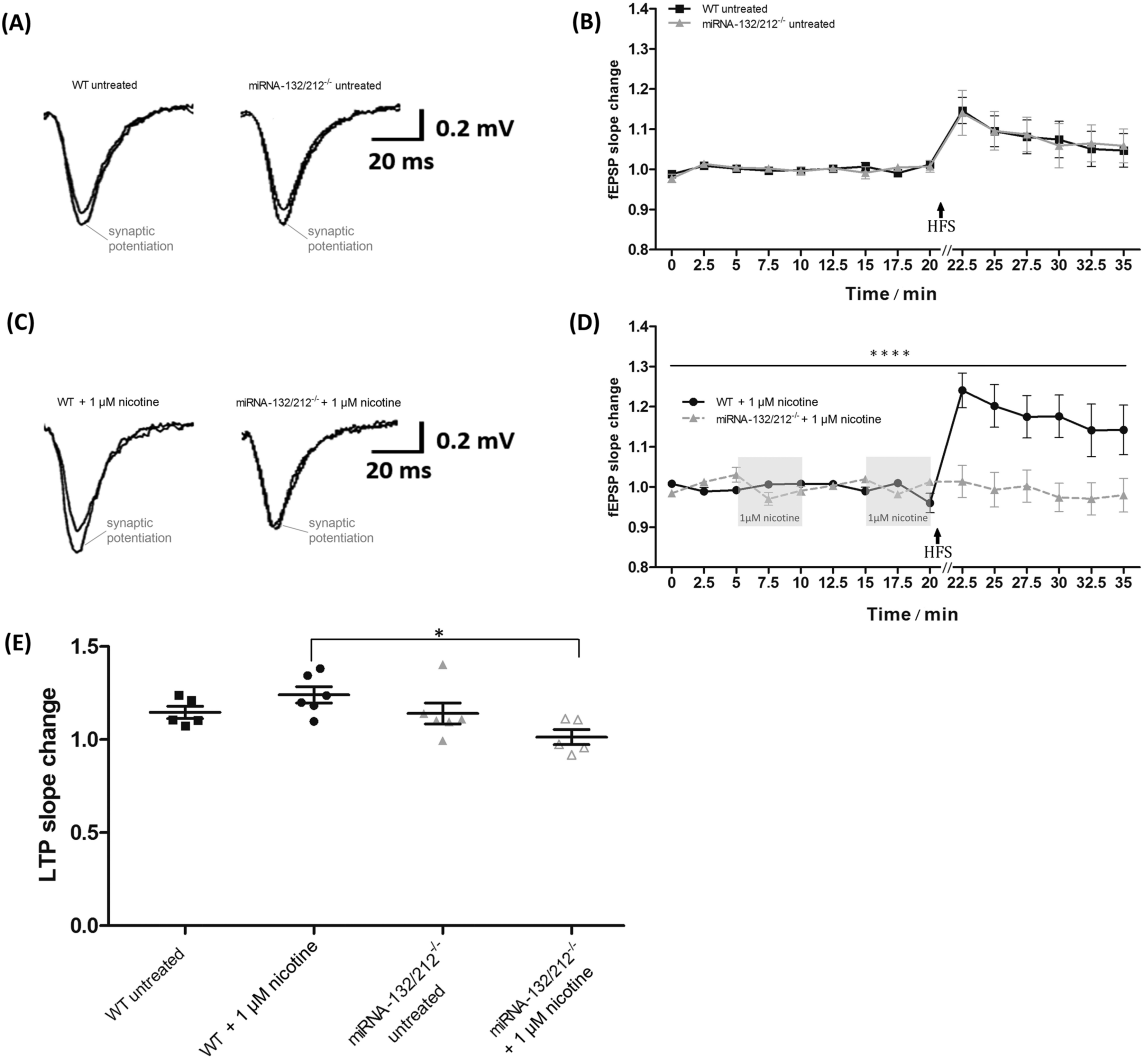
Nicotine stimulation abolishes synaptic potentiation in hippocampal dentate gyrus of miRNA‐132/212 gene knockout. A, Representative traces of field excitatory postsynaptic potentials (fEPSPs), recorded at baseline conditions and after the application of potentiation‐inducing protocols (Section 2), from untreated hippocampal slices derived from untreated wild‐type controls and untreated miRNA‐132/212^−/−^ mice. The responses generated after delivery of electrical stimulation were taken at 22.5 min and are indicated as “synaptic potentiation” in the graph. B, Temporal course of the changes in fEPSPs slopes as recorded before and after the application of potentiation‐inducing protocols in hippocampal slices from untreated wild‐type and untreated miRNA‐132/212^−/−^ mice. No statistically significant differences were observed between the groups. C, Representative traces of fEPSPs, recorded at baseline conditions and after the application of potentiation‐inducing protocols, from untreated hippocampal slices derived from untreated wild‐type controls and untreated miRNA‐132/212^−/−^ mice. The responses generated after delivery of electrical stimulation are indicated as “synaptic potentiation” in the graph and were obtained after the dual application of 1‐μM nicotine, which was delivered during baseline conditions as described below. D, Temporal course of the changes in fEPSPs slopes as recorded before and after the application of potentiation‐inducing protocols in hippocampal slices from untreated wild‐type (*n* = 6) and untreated miRNA‐132/212^−/−^ (*n* = 5) mice. Two consecutive pulses of 5‐min exposure to 1‐μM nicotine, spaced by a 5‐min washout period, preceded the delivery of the application of potentiation‐inducing protocols as illustrated in the figure in the shadowed box insets. A pronounced and statistically significant enhancement of synaptic potentiation was observed in recordings from wild‐type animals, whereas a virtual abolishment of the potentiation response was detected in slices from miRNA‐132/212^−/−^ mice. E, Scatter plots showing the field responses after delivering potentiation‐inducing protocols in all four investigated groups at 22.5 min. The high‐frequency stimulation protocol used to induce potentiation was delivered right after the recording obtained at 20 min as indicated in the figure (HFS + arrow). Data are presented as mean ± SEM. Please see details for the statistics on the main text

While regulation of neuroplasticity functions by miRNAs activity has been previously proposed,^100^, ^101^, ^102^ little continue to be known about the influence of miRNAs modulation on the effects of addictive substances on the dentate gyrus, a brain structure critical for memory formation and maintenance.^103^, ^104^, ^105^ We therefore here examined, in hippocampal slices, the effects of nicotine stimulation on dentate gyrus synapses in hippocampal slices from WT and miRNA‐132/212^−/−^ mice. Both groups of slices were subjected to electrical stimulation protocols that are known to induce transitory forms of synaptic strengthening in hippocampal slices in murine animal models.^80^, ^106^, ^107^, ^108^ Recordings from slices obtained from both WT and miRNA‐132/212^−/−^ presented with transitory forms of synaptic strengthening that were not significantly different under untreated control conditions (Figure 3A,B). However, two consecutive exposures to 1‐μM nicotine stimulation delivered with a 5‐min interval resulted in a robust increase in synaptic strengthening in slices from WT animals, whereas an opposite response was observed in slices from miRNA‐132/212^−/−^ mice, where synaptic potentiation was virtually abolished (Figure 3C,E). Mixed‐effects ANOVA analysis showed a significant effect of time (^****^ *P* < 0.0001, *F*_14,140_ = 13.02), no significant effect of the genotype (^ns^ *P* = 0.1975, *F*_1,10_ = 1.906), no significant genotype × treatment interaction (^ns^ *P* = 0.1926, *F*_1,10_ = 1.952) and highly significant time × genotype × treatment interaction (^****^ *P* < 0.0001, *F*_14,110_ = 4.085; Figure 3C,D). Two‐way ANOVA of the 22.5 min values showed no significant effect of treatment (^ns^ *P* = 0.7234, *F*_1,18_ = 1.292) and significant effect of genotype (^*^ *P* = 0.02, *F*_1,18_ = 6.486) and genotype × treatment interaction (^*^ *P* = 0.03, *F*_1,18_ = 5.863). Bonferroni post hoc analysis found significant differences between WT treated and miRNA‐132/212^−/−^ treated slices (^*^ *P* = 0.02, *t*_18_ = 3.513) and no significant differences between other groups (WT vs. WT treated: ^ns^ *P* = 0.9725, *t*_18_ = 1.458; miRNA‐132/212^−/−^ vs. miRNA‐132/212^−/−^ treated slices: ^ns^ *P* = 0.3893, *t*_18_ = 1.966; WT vs. miRNA‐132/212^−/−^: ^ns^ *P* > 0.999, *t*_18_ = 0.0888; Figure 3E).

### 3.3 Nicotine stimulation promotes dentate gyrus synaptic depression in miRNA‐132/212^−/−^ mouse

**FIGURE 4:**
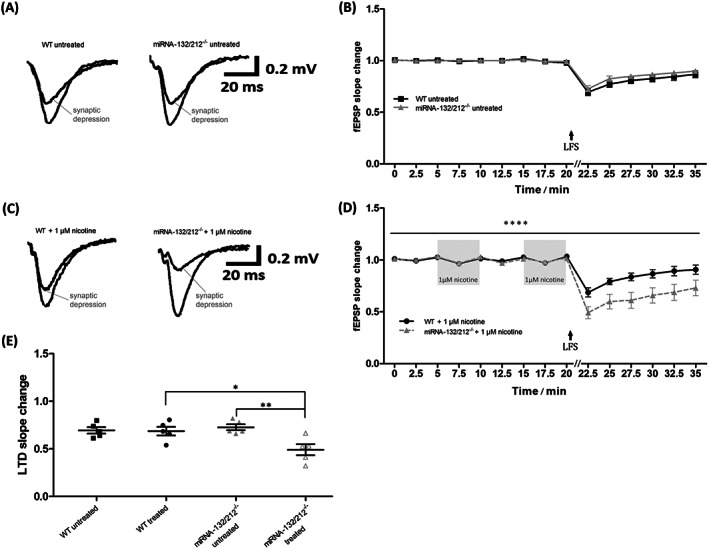
Nicotine stimulation promotes synaptic depression in hippocampal dentate gyrus of miRNA‐132/212 gene knockout. A, Representative traces of field excitatory postsynaptic potentials (fEPSPs), recorded at baseline conditions and after the application of synaptic depression‐inducing protocols, from untreated hippocampal slices derived from untreated wild‐type controls and untreated miRNA‐132/212^−/−^ mice. The responses generated after delivery depression‐inducing electrical stimulation were taken at 22.5 min and are indicated as “synaptic depression” in the graph. B, Temporal course of the changes in fEPSPs slopes as recorded before and after the application of depression‐inducing protocols in hippocampal slices from untreated wild‐type and untreated miRNA‐132/212^−/−^ mice. No statistically significant differences were observed between the groups. C, Representative traces of fEPSPs, recorded at baseline conditions and after the application of depression‐inducing protocols, from untreated hippocampal slices derived from untreated wild‐type controls and untreated miRNA‐132/212^−/−^ mice. The responses generated after delivery of electrical stimulation are indicated as “synaptic depression” in the graph and were obtained after the dual application of 1‐μM nicotine, which was delivered during baseline conditions as described below. D, Temporal course of the changes in fEPSPs slopes as recorded before and after the application of depression‐inducing protocols in hippocampal slices from untreated wild‐type and untreated miRNA‐132/212^−/−^ mice. Two consecutive pulses of 5‐min exposure to 1‐μM nicotine, spaced by a 5‐min washout period, preceded the delivery of the application of potentiation‐inducing protocols as illustrated in the figure in the shadowed box insets. While slices from wild‐type (n = 5) and untreated miRNA‐132/212^−/−^ (*n* = 5) mice presented both with synaptic depression responses, synaptic depression was however statistically much more pronounced in the miRNA‐132/212^−/−^ group. E, Scatter plots showing the field responses after delivering synaptic depression‐inducing protocols (Section 2) in all four investigated groups at 22.5 min (2.5 min poststimulation). Mean and SEM are indicated by the black horizontal lines; error bars are presented as SEM. The low‐frequency stimulation protocol used to induce depression was delivered right after the recording obtained at 20 min as indicated in the figure (LFS + arrow). Data are presented as mean ± SEM. Please see details for the statistics on the main text

Both LTP and LTD have been proposed as putative mechanisms participating in learning and memory processes in vivo,^109^, ^110^ and abundant literature have also associated both LTP and LTD with the brain neurobiology of drug addiction.^111^, ^112^, ^113^, ^114^, ^115^, ^116^, ^117^, ^118^, ^119^, ^120^, ^121^ Nevertheless, the involvement of miRNA‐132/21 in the regulation of both LTD and the effects of nicotine on the neurogenic dentate gyrus remained uncharacterized. We therefore next obtained hippocampal slices from miRNA‐132/212^−/−^ and WT mice and examined the properties of LTD (Section 2) and of nicotine stimulation on dentate gyrus synapses. Under untreated control conditions, field potential recordings in slices obtained either from WT or from miRNA‐132/212^−/−^ presented with synaptic depression responses that were statistically indistinguishable between the two groups (Figure 4A,B). However, while no statistically significant effects of the nicotine treatment was observed in slices obtained from the WT controls after the treatment with the 5‐min paired exposure to 1‐μM nicotine, a pronounced statistically significant promotion of synaptic depression was apparent in slices obtained from miRNA‐132/212^−/−^ (Figure 4C,E). Mixed‐effects ANOVA analysis showed a significant effect of time (^****^ *P* < 0.0001, *F*_14,112_ = 113.60), no significant effect of the genotype (^ns^ *P* = 0.1309, *F*_1,8_ = 2.832), significant effect of the treatment (^*^ *P* = 0.04, *F*_1,8_ = 6.316), significant genotype × treatment interaction (^*^ *P* = 0.04, *F*_1,8_ = 6.297) and highly significant time × genotype × treatment interaction (^****^ *P* < 0.0001, *F*_14,112_ = 4.895; Figure 4C,D). Two‐way ANOVA of the 22.5‐min values showed significant effect of treatment (^*^ *P* = 0.02, *F*_1,16_ = 17.906) and no significant effect of genotype (^ns^ *P* = 0.0766, *F*_1,16_ = 3.584) and significant genotype × treatment interaction (^*^ *P* = 0.02, *F*_1,16_ = 6.986). Bonferroni post hoc analysis found significant differences between WT treated and miRNA‐132/212^−/−^ treated slices (^*^ *P* = 0.04, *t*_16_ = 3.208) and miRNA‐132/212^−/−^ versus miRNA‐132/212^−/−^ treated slices (^**^ *P* = 0.009, *t*_16_ = 3.857) and no significant differences between other groups (WT vs. WT treated: ^ns^ *P* > 0.999, *t*_16_ = 0.1192; WT vs. miRNA‐132/212^−/−^: ^ns^ *P* > 0.999, *t*_16_ = 0.5302; Figure 4E).

### 3.4 miRNA‐132/212 deletion alters protein levels of α7‐nAChR and pERK in mouse hippocampus

**FIGURE 5:**
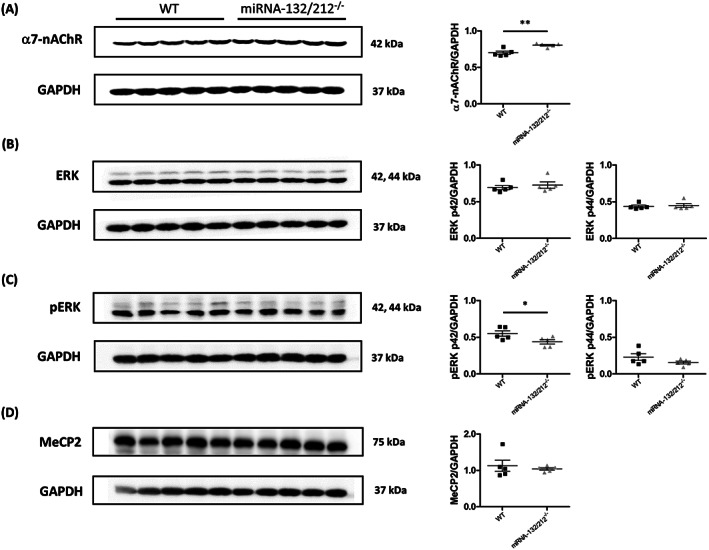
The miRNA‐132/212 deletion alters protein levels of α7‐nAChR and pERK in mouse hippocampus. Total cell lysates from male mouse bilateral hippocampi were analysed by Western blot to determine the protein expression levels of α7‐nAChR, ERK and pERK. A, Representative immunoblots and relative levels of α7‐nAChR protein together with corresponding GAPDH levels, indicating significant increase in protein levels of α7‐nAChR in the hippocampus of miRNA‐132/212^−/−^ mice, compared with wild‐type mice. B, Representative immunoblots and relative levels of ERK1 (p44) and ERK2 (p41) protein together with corresponding GAPDH levels in the hippocampus of miRNA‐132/212^−/−^ mice. Analysis showed no changes in protein levels of ERK compared with wild‐type mice. C, Representative immunoblots and relative levels of pERK and GAPDH levels indicating significant decrease in protein levels of pERK in the hippocampus of miRNA‐132/212^−/−^ mice compared with wild‐type mice. No significant differences were observed in protein levels of pERK. D, Representative immunoblots and relative levels of MeCP2 protein together with corresponding GAPDH levels, indicating no significant differences in protein levels of MeCP2 in the hippocampus of miRNA‐132/212^−/−^ mice, compared with wild‐type mice. Results are shown relative to GAPDH. Data are expressed as mean ± SD. *n* = 5 per group. WT, wild type; kDa, kilodalton. Please see details for the statistics on the main text

Abundant scientific literature has recently started to emerge describing miR‐132 as a strong regulator of members of the cholinergic signalling pathway in several different tissues.^122^, ^123^, ^124^, ^125^, ^126^, ^127^, ^128^, ^129^, ^130^ Virtually no information was available, however, about the effects of miRNA‐132/212^−/−^ gene disruption on the expression levels of acetylcholinergic receptors in the mammalian neurogenic dentate gyrus, a structure widely demonstrated to be importantly involved in the neurobiology of nicotine drug addiction.^131^, ^132^, ^133^, ^134^, ^135^, ^136^, ^137^, ^138^, ^139^ On the grounds of the above‐described effect of nicotine on the properties of dentate gyrus synaptic potentiation and synaptic depression in miRNA‐132/212^−/−^ mice slices, we next explored the impact of miRNA‐132/212 gene disruption on the protein expression levels of α7‐nAChR, total ERK and phosphorylated ERK (pERK), as well as for the previously proposed target of miR‐132 CpG binding protein MeCP2. Western blot analyses of cell lysates derived from bilateral hippocampi obtained from WT controls and miRNA‐132/212^−/−^ mice indicated a previously uncharacterized, statistically significant increase in the levels of the alpha‐7 nicotinic acetylcholine receptor (α7‐nAChR) in the hippocampi of miRNA‐132/212^−/−^ mice (Figure 5) compared with their related WT control littermates (^**^ *P* = 0.004; *t*_8_ = 4.153; *t* test). On the contrary (Figure 5), a statistically significant reduction in the levels of the protein of p42 pERK was detected in miRNA‐132/212^−/−^ mice compared with their related WT controls (p42 ^*^ *P* = 0.0452; *t*_8_ = 2.371; p44 ^ns^ *P* = 0.1605; *t*_8_ = 1.518; *t* test), whereas no differences were observed for the protein expression levels of total ERK (p42 ^ns^ *P* = 0.5388; *t*_8_ = 0.6420; p44 ^ns^ *P* = 0.6825; *t*_8_ = 0.4244; *t* test) and MeCP2 (^ns^ *P* = 0.6007; *t*_8_ = 0.5449; *t* test; Figure 5).

## 4 DISCUSSION

### 4.1 Combining pharmacological and electrophysiological approaches to study the effects of nicotine on neuronal plasticity

Studies of the properties of synaptic plasticity in experimental settings both in vivo and ex vivo comprise a powerful tool to examine the functional signalling mechanisms likely responsible for the changes in the neuronal circuits during learning and memory processes.^140^, ^141^, ^142^, ^143^, ^144^, ^145^, ^146^, ^147^, ^148^, ^149^, ^150^ Increasing reports show that electrical‐stimulation protocols (like those used to induce LTP) recapitulate many morphofunctional phenomena occurring at synapses in vivo during memory formation.^83^, ^88^, ^108^, ^141^, ^151^, ^152^, ^153^, ^154^, ^155^, ^156^, ^157^ Some behavioural learning paradigms in murine animal models can further trigger comparable protein‐level enhancements or increase in field‐potential amplitudes similar to the delivery of LTP‐inducing high‐frequency pulses of electrical stimulation in slices.^158^ In fact, pioneering reports from experiments in vivo have demonstrated the presence of learning‐dependent changes in synaptic strength in hippocampal circuits.^147^, ^150^, ^159^ These observations thus validate the implementation of this strategy as experimental model in the search for the molecular/functional mechanisms underlying memory storage. Using comparable approaches, we here examined the effects of miR‐132/212 gene disruption on neuroplasticity and on the response to nicotine. We provide the first molecular‐biochemical and functional experimental evidence proposing miR‐132/212 as a molecular element influencing the effects of nicotine on the neuroplasticity responses in the mouse dentate gyrus.

### 4.2 A role for miR‐132/212 in the regulation of nicotinergic signalling in the hippocampal dentate gyrus

The hippocampal dentate gyrus is a brain region necessary for proper learning and memory functions^160^, ^161^, ^162^, ^163^, ^164^, ^165^ that plays critical roles in the pathophysiology of drug addiction.^116^ Nicotine is a widespread substance of abuse capable of causing profound alterations to the hippocampus.^131^, ^132^, ^133^, ^134^, ^135^, ^136^, ^137^, ^138^, ^139^ miR‐132/212 modulates a wide variety neuronal processes^22^, ^37^, ^38^, ^125^, ^166^, ^167^, ^168^, ^169^ including hippocampal synaptic plasticity and memory‐related functions.^22^, ^23^, ^38^, ^167^ The involvement of miR‐132/212 in the regulation of the effects of nicotine in the hippocampal dentate gyrus had remained however unexplored. Our results indicate that the absence of miR‐132/212 results in a previously uncharacterized acute and bimodal synaptic response to nicotine, as reflected in a pronounced enhancement of synaptic depression and a virtual abolishment of synaptic potentiation.

α7‐nAChRs are of chief relevance for memory‐related synaptic plasticity and are involved in the addiction to nicotine.^62^, ^63^, ^64^, ^65^ Our experimental observations are therefore of relevance for different scientific disciplines, including neuroscience, biochemistry, molecular biology and neuropharmacology and basic and clinical research: miRNAs are powerful regulators of the gene expression^1^, ^2^, ^3^, ^4^, ^5^, ^6^, ^7^, ^9^, ^10^ that play key roles in the modulation of the neuronal function of many different species.^14^, ^17^, ^18^, ^19^, ^20^ miRNAs also influence several vital neurodevelopmental and adult brain neuronal processes.^14^, ^18^, ^19^, ^21^ Moreover, alterations of the miRNAs activity is associated with Alzheimer's^27^, ^28^, ^29^, ^30^, ^31^, ^32^, ^33^, ^34^, ^35^ and Parkinson's diseases.^11^ Understanding of the miRNAs systems of neuronal regulation is thus timely and relevant. We postulate that miR‐132/212 gene disruption results, either directly or indirectly, in altered levels of α7‐nAChR, which results in a bimodal response to nicotine that affects both synaptic depression and strengthening through mechanism that still remain to be characterized.

### 4.3 Putative direct/indirect molecular elements linking miR‐132/212 down‐regulation and nicotinergic signalling

Nicotine facilitates the release of Ach, which plays an important role in cognitive functions^170^, ^171^ and facilitates LTP induction by interacting with the nAChRs subtypes α7 in the hippocampus.^172^ In line with these previously described observations, we detected enhanced synaptic transmission (potentiation) upon high‐frequency electrical stimulation (known to induce LTP) in WT mice that were previously exposed to nicotine, effects that were abolished in miR‐132/212 KO mice. Furthermore, our protein analysis for the first time describes that miR‐132/212 gene disruption results in a marked enhancement in the protein expression levels of the α7‐nAChR paralleled by a pronounced decrease of pERK, with no changes in total ERK or MeCP2. Interestingly, while MeCP2 has been proposed as a target of miR‐132 in neurological disorders like Autism spectrum disorder (ASD) and Huntington and Parkinson's diseases,^173^ several studies reported unaltered levels of MeCP2 protein upon miR‐132 up‐regulation or down‐regulation,^38^, ^174^ a finding consistent with our observation, thus suggesting the existence of other alternative mechanisms regulating MeCP2 in the pathophysiology apart of miR‐132/212. On the other hand, the absence of alterations in levels of total ERK1/2 accompanied by a decrease in pERK1 indicates dynamic and highly phosphorylation‐specific alterations of ERK1/2 protein expression by dysregulated miR‐132/212 cluster. Our observations are thus in line with previous studies that proposed the existence of a homeostatic feedback loop between the miR‐132/212 cluster and Mapk/ERK pathway.^175^, ^176^, ^177^ Specifically, down‐regulated levels of miR‐132‐3p, human homologous and a significant part of miR‐132/212 cluster, have been proposed to have a delayed effect on the Mapk1/ERK2 protein levels.^175^ MAPK signalling can mediate miRNAs biogenesis through their ability to stabilize the Dicer enzyme by regulating the TRBP (HIV‐1 TAR RNA‐binding protein) binding^178^, ^179^, ^180^ and thus may play an important role in the changes in gene expression post‐LTP induction.

Our observations therefore invite further research to verify independently our findings and to contribute elucidating the mechanisms by which miR‐132/212 might, directly or indirectly, influence α7‐nAChR and pERK expression and the effects of nicotine on hippocampal neuroplasticity. The targeting of members of the acetylcholinergic signalling pathway by the miR‐132/212 family of miRNAs is not without precedents in the scientific literature. Several other molecular elements could mediate in the effects of miR‐132/212 gene disruption on the neuroelectrical plasticity responses of hippocampal synapses to nicotine. For example, acetylcholinesterase (AChE) is an enzyme involved in the functional cessation of the residual activity of neuronal synaptic transmission mediated by Ach and likewise implicated in Alzheimer's disease.^181^ Previous reports have described that AChE is capable of acting as a modulator of the activity of α7‐nAChRs.^182^ More importantly, AChE is indeed a target of miRNAs from the miR‐132/212 family.^122^, ^123^, ^126^, ^127^, ^128^ Additionally, disruption in the proper targeting and inhibition of AChE expression by miR‐132 (due to the down‐regulation it this miRNA) have been further proposed to be causally linked with the progression of dementia after ischaemic stroke.^130^ Recent experiments have also described that inhibitors of the AChEs can act to promote neuroprotection or neurogenesis via the activation of α7‐nAChR thus resulting in an increase in the levels of growth factors in the mouse hippocampus.^183^ Indeed, compounds acting as inhibitors of AChEs have been clinically used for the treatment of Alzheimer's disease.^184^ The targeting of AChE by miR‐132 suggest thus that AChE could indirectly mediate in the here described effects of miR‐132/212 gene disruption on the neuroplasticity responses to nicotine in the dentate gyrus, a hypothesis requiring future research for clarification.

### 4.4 Conclusions

The specific molecular mechanisms linking the action of drugs of abuse with brain neuroplasticity and memory storage remain elusive, and consequently, clinical interventions continue to fail to alter drug‐dependence‐related behaviours, particularly in the case of nicotine addiction. ACh affects the hippocampus due to its selective effect on episodic and semantic memory formation.^185^ Therefore, the expression and correct regulation of ACh synthesis, its release, and the properties of its effect on specific receptors are likely key factors in preserving and enhancing memory‐related neural functions during drug addiction in the mammalian nervous systems. Interestingly, the overexpression of specific miRNAs in the CA1 hippocampal region (an area also of central importance for spatial memory) has been shown to alter some long‐term forms of synaptic plasticity.^38^, ^186^ Moreover, in the hippocampus, the prevalent subunit subtypes are the α7 and α4β2 subtypes, with α7‐nAChRs having the highest density in the pyramidal neurons, localized presynaptically and postsynaptically.^187^ Dysfunction or deactivation of those receptors has shown to lead to various serious neuropathologies including Alzheimer's disease,^181^, ^188^ perhaps the most debilitating and widespread form of memory dysfunction. It is noteworthy to highlight that whereas data presented here indicate that miRNA‐132/212^−/−^ mice show an increase in the levels of alpha‐7 nicotinic Ach receptors, they however have a decreased response to nicotine administration (to LTP) compared with controls, thus suggesting that inhibitory mechanism has been promoted, including either enhancing of reversal of LTP (depotentiation; see also Fujii and Sumikawa^52^) or promotion of LTD as indicated here by nicotine‐enhanced LTD in miRNA‐132/212^−/−^ KO animals). The molecular mechanisms responsible for these rather paradoxical effects remain unclear, and the capability of nicotine to induce a large variety of different actions is surely determined by its capability to interact with diverse nicotinic Ach receptor subtypes. For example, whereas some authors have described that nicotine can enhance LTP,^189^, ^190^ other authors have described that nicotine can act as both synaptic enhancer and depressor depending on the levels of A2 nicotinic Ach receptors and in a pathway‐specific manner.^191^ Nicotine (3 mg/kg, i.p.) delivered 1 h prior to the LTP induction has been also shown not to enhance LTP recorded in vivo but rather to exacerbate the impairments of LTP induced by Aβ_1–40_ treatment.^192^ In slices, nicotine can also fail to induce synaptic potentiation in CA1 pyramidal neurons of AD11 antinerve growth factor transgenic mice in a manner related to the Aβ levels.^193^ Interestingly, a recent manuscript has described that the deficiency in miRNA‐132/212 results in enhanced levels of Aβ production in mouse models of Alzheimer's disease.^194^ These observations thus also suggest that the deficiency of nicotine to positively influence LTP in our experiments can be related to the effects of miRNA‐132/212 gene deletion on the levels of Aβ, a hypothesis currently under investigation in our laboratory. Nicotine appears thus to have the capability to exert bimodal effects, inducing either inhibition or strengthening of synaptic responses on the same circuits. Interestingly, it has been demonstrated that stimulation with the parasympathomimetic choline ester carbachol (CCh), which can simultaneously act as a nicotinic and muscarinic Ach receptor agonist, can result in a bimodal effect either suppressing or potentiating the synaptic responses depending upon the centration and duration of CCh exposure,^43^ a bimodal process that could therefore be influenced by miRNA regulation. Further experiments, including necessary controls examining the effects of muscarinic receptor activation in miR‐132/212 KO mice, are therefore required to clarify the possible functional crosstalk between Ach receptor‐mediated signalling and miR‐132/212 regulation in the hippocampal synaptic function.

Future experiments from ours and/or others are also required to rule out whether α7‐nAChRs become direct targets of miR‐132/212 under specific frequency‐dependent stimulation conditions. Similarly, experiments using acetylcholinesterase inhibitors like galantamine or *Tabernaemontana divaricata* extract^184^ could shed light into the signalling mechanisms underlying the here described effect of nicotine on synaptic potentiation and depression in the hippocampus of miR‐132/212^−/−^ mice. Together, all these observations suggest a potential participation of miR‐132/212 in nicotinergic signalling in the mammalian hippocampus, a brain structure importantly involved in the neurobiology of drug addiction.

## AUTHORS CONTRIBUTIONS

FM conceived and directed the project. FM and TS designed experiments and conducted electrophysiological experiments and data analyses. HB, AA and DB replicated electrophysiological experiments and contributed to data analyses. TS and FM wrote the manuscript. All authors discussed the findings and approved the manuscript.

## DISCLOSURE/CONFLICT OF INTEREST

The authors report no conflicts of interest.

## Data Availability

The datasets generated for this study are available on request to the corresponding author.
